# High-yield BMSC-derived exosomes by the 3D culture system to enhance the skin wound repair

**DOI:** 10.1093/rb/rbaf022

**Published:** 2025-04-10

**Authors:** Jie Wu, Siqi Li, Hao Wang, Yuanbo Qi, Sheng Tao, Peifu Tang, Daohong Liu

**Affiliations:** Medical School of Chinese PLA, Beijing 100853, China; Department of Orthopedics, The First Medical Center of Chinese PLA General Hospital, Beijing 100853, China; Senior Department of Orthopedics, The Fourth Medical Center of PLA General Hospital, Beijing 100142, China; Department of Orthopedics, The Eighth Medical Center of Chinese PLA General Hospital, Beijing 100091, China; Department of Nephrology, The Second Medical Center of Chinese PLA General Hospital, Beijing 100853, China; Department of Orthopaedics, Lanzhou University Second Hospital, Lanzhou, Gansu 730030, China; Department of Orthopedics, The Eighth Medical Center of Chinese PLA General Hospital, Beijing 100091, China; Senior Department of Orthopedics, The Fourth Medical Center of PLA General Hospital, Beijing 100142, China; Department of Orthopedics, The Eighth Medical Center of Chinese PLA General Hospital, Beijing 100091, China; Medical School of Chinese PLA, Beijing 100853, China; Senior Department of Orthopedics, The Fourth Medical Center of PLA General Hospital, Beijing 100142, China; Medical School of Chinese PLA, Beijing 100853, China; Senior Department of Orthopedics, The Fourth Medical Center of PLA General Hospital, Beijing 100142, China

**Keywords:** mesenchymal stem cells, three-dimensional culture system, exosomes, wound healing

## Abstract

Wound defects pose a substantial challenge in clinical practice, often resulting in prolonged healing times and an elevated risk of infection. Insufficient vascularization is a critical factor that adversely affects wound healing. Exosomes obtained from bone mesenchymal stem cells (BMSC-exos) have demonstrated significant promise in accelerating tissue repair by promoting angiogenesis. However, their limited yield and suboptimal biological functions impede widespread clinical application in enhancing wound healing. Prior research has indicated that 3D cultures can boost exosome secretion when compared to conventional 2D cultures. However, the currently prevalent 3D culture methods often necessitate expensive equipment or cumbersome procedures. This study investigates a cost-effective and user-friendly 3D culture system developed using gelatin methacrylate (GelMA). Our findings indicate that a 5% concentration of GelMA provides an optimal environment for the 3D culture of BMSCs. Furthermore, we observed that 3D culture significantly delays the senescence of BMSCs, thereby creating favorable conditions for the sustained production of exosomes. Additionally, 3D cultivation has the potential to boost exosome secretion and enhance their angiogenic capabilities. *In vivo* experiments further confirmed that BMSC-exos from a 3D environment exhibit enhanced capabilities to promote wound healing. These results suggest that GelMA-based 3D cultures offer a novel strategy for both industrial production and clinical application of exosomes.

## Graphical abstract

**Figure:**
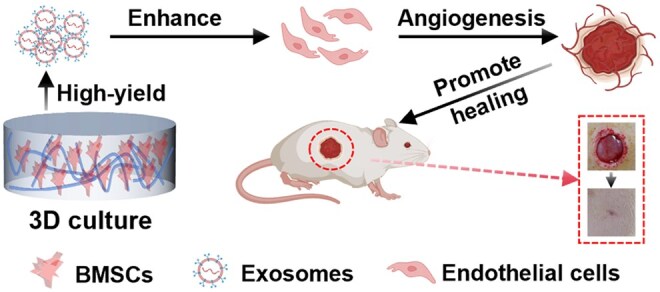

## Introduction

The skin serves a vital function in the human body, encompassing providing mechanical protection, preventing moisture loss, regulating body temperature and detecting external stimuli. Skin defects caused by trauma or disease present a common clinical challenge, often leading to dehydration, electrolyte imbalance, and protein loss, thereby increasing susceptibility to infection [1, 2]. Impaired wound healing significantly affects the quality of life for patients, leading to considerable challenges that not only affect the individuals directly but also impose significant burdens on society as a whole [3]. Wound healing represents a complex and systematic physiological process [4–6]. When the wound is excessively large [7] or influenced by pathological factors [8], it may pose challenges for healing [9]. Facilitating wound healing using appropriate methodologies is a primary objective for clinical practitioners. Currently, the commonly used methods for promoting wound healing in clinical practice encompass a variety of dressings, vacuum sealing drainage, bioengineered tissue substitutes, and autologous skin grafts. Each of these strategies presents distinct advantages as well as inherent limitations [9–11].

Bone mesenchymal stem cells (BMSCs) have become essential progenitor cells in the domain of tissue engineering, owing to their exceptional capabilities in tissue repair [12]. An expanding body of research suggests that exosomes secreted by BMSCs (BMSC-exos) are capable of performing most of the functions of BMSCs. They are non-immunogenic and non-tumorigenic, offering the advantages of convenience and processability [13]. A crucial element of the wound-healing process is angiogenesis, with inadequate angiogenesis being a common cause of many non-healing wounds [14–16]. Due to their role in promoting angiogenesis, BMSC-exos exhibit significant potential for application in enhancing wound healing [6, 17]. However, the limited yield and insufficient repair capacity of BMSC-exos pose significant constraints on their clinical translation [18].

While numerous approaches have been identified to enhance the yield of exocrine secretions and strengthen their functions, concerns regarding safety and operability still persist. Traditionally, cell culture has been conducted in a 2D setting, which significantly diverges from the physiological conditions of cell growth. In contrast, 3D culture more accurately replicates the morphology, behavior, and function of cells under physiological states [19]. The research conducted by Zhu *et al*. [20] illustrates that 3D culture markedly improves the production of exosomes, and the exosomes generated through 3D culture exhibit superior angiogenic capability, leading to enhanced promotion of wound healing. The research conducted by Yuan *et al*. [21] reveals notable variations in the composition of miRNA and functional proteins between 3D-exos and 2D-exos. This discrepancy may explain the enhanced repair capabilities observed in 3D-exos. Furthermore, evidence has demonstrated that 3D culture can significantly mitigate senescence-related alterations in MSCs [22], which could be advantageous for sustained exosome production. It is noteworthy that there are currently various 3D cell culture techniques available. Although each of these methods has its own advantages, they may exhibit limitations in the large-scale production of exosomes [19]. For instance, bioreactor methods require expensive equipment and carry an increased risk of cellular contamination. Additionally, low-adhesion spheroid culture methods struggle to ensure uniformity in spheroid size, which can lead to heterogeneity in the produced exosomes. Furthermore, hanging drop culture presents challenges during the exosome collection process. Synthetic hydrogels demonstrate exceptional performance in replicating the cellular microenvironment under physiological conditions, serving as crucial tools for investigating cell responses to various physicochemical signals within 3D culture systems [23–24]. Moreover, their ease of mass production and stable physicochemical properties make them well-suited for large-scale 3D cell culture. Gelatin methyl acryloyl (GelMA) is a type of synthetic biological macromolecule with excellent biocompatibility and formability [25]. The commercialization of UV-curable GelMA has been widely applied in various research applications [26]. Therefore, employing the GelMA-based 3D culture technique, which offers cost-effectiveness and ease of operation, holds promise as a method for exosomes production. However, current research predominantly concentrates on the application of GelMA as a carrier for exosomes. There is a notable deficiency in comprehensive studies investigating the three-dimensional culture of BMSCs utilizing GelMA and its subsequent effects on exosome secretion.

**Figure 1.**
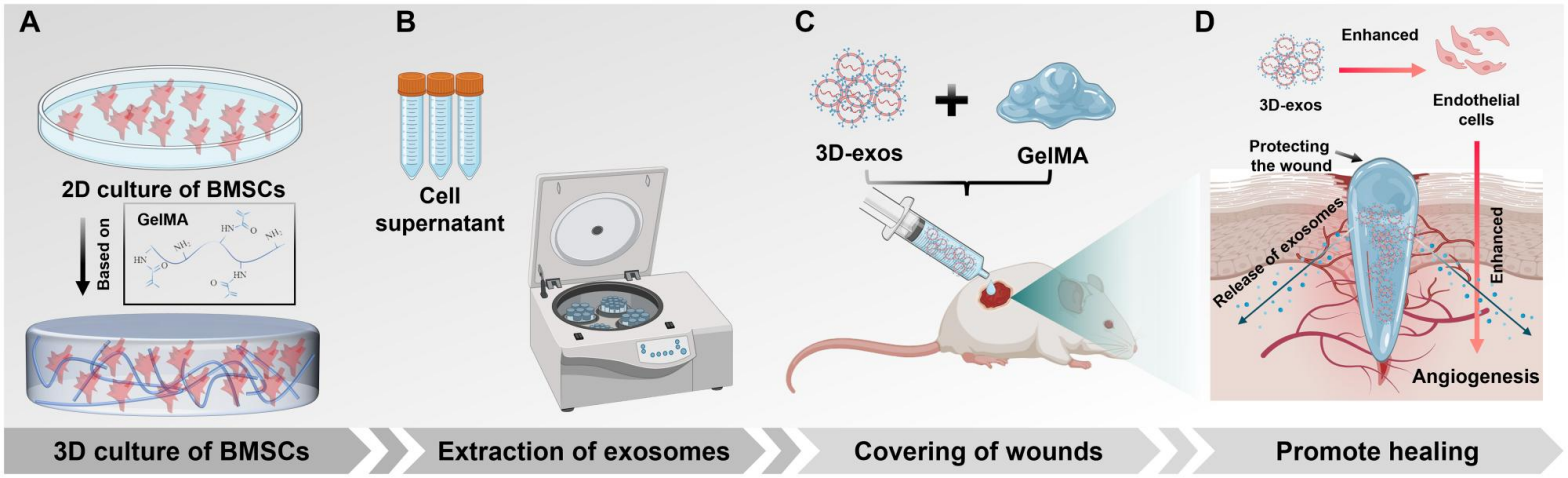
Schematic illustration of the primary processes involved in this study. (**A**) Construction of a 3D culture system based on GelMA. (**B**) Extraction of exosomes from cell culture supernatant. (**C**) Cover the wound with a hydrogel containing BMSC-exos. (**D**) By encouraging blood vessel proliferation, the hydrogel containing BMSC-exos promotes wound healing and has a protective effect. Image created with BioRender.com, with permission.

In this study, we investigated the optimal GelMA concentration for 3D culture of BMSCs and assessed the impact of this 3D culture environment on the secretion of BMSC-exos. Furthermore, we assessed the pro-angiogenic potential of BMSC-exos under different culture dimensions. Subsequently, we utilized a rat wound healing model to demonstrate that BMSC-exos derived from 3D culture exhibited an enhanced ability to facilitate wound healing. Finally, we conducted transcriptome sequencing of cells in different culture dimensions to preliminarily elucidate the mechanism underlying the promotion of exosome secretion by 3D culture (Figure 1).

## Methods

### 2D and 3D culture of BMSCs

The BMSCs derived from SD rats (RASMX-01001) were obtained from Cyagen Biotechnology Company (Guangzhou, China). These cells were cultured in Dulbecco’s Modified Eagle Medium (DMEM), which was sourced from Gibco Biotechnology Company (NY, USA). The culture medium was enriched with 10% fetal bovine serum, also from Gibco, and supplemented with 1% penicillin-streptomycin obtained from Solarbio in Beijing, China. The cultures were maintained at 37°C in a humidified atmosphere containing 5% CO_2_. 3D cultures were performed using GelMA-30 (Engineering For Life, Zhejiang, China) at concentrations of 5% w/v (G5 group), 10% w/v(G10 group) and 15% w/v (G15 group). The hydrogel was dissolved by immersion in PBS containing the photoinitiator lithium phenyl-2,4,6-trimethylbenzoylphosphinate (LAP, 0.25% w/v) with agitation. After filtration through a 0.22-μm filter (Millipore, USA), BMSCs were resuspended within the hydrogel to attain a cell density of roughly 1.0 × 10^6^ cells/ml. Each well in a 24-well plate received 300 μl of cell-containing hydrogel, which was then cured for 30 seconds by 405 nm UVR irradiation. The cells were then put in the incubator to be cultivated after 1 ml of culture media had been supplied to each well.

### Cell counting kit-8 assay and live/dead viability assay

Cell proliferation was evaluated using the Cell counting kit (CCK)-8 kit (Dojindo, Japan). During the first, fourth and seventh days of cell culture, the original medium was replaced with a complete medium containing 10% CCK-8. Following a 2-hour incubation in the dark inside an incubator, the medium was moved to a 96-well plate for further analysis. The absorbance was measured at a wavelength of 450 nm using enzyme labeling techniques. To enhance the reliability of the results, three replicate wells were employed for each condition, and the entire experiment was conducted in triplicate.

The cell viability of BMSCs in hydrogels with various concentrations was detected using the live/dead staining kit (Abbkine, China). On the first, fourth and seventh days of the cell culture period, the hydrogels containing cells underwent three washes with PBS. Following the provided instructions, LiveDye/NucleiDye reagents were introduced into each well. After a 30-minute incubation, the samples were washed once more prior to observation under a fluorescence microscope (Nikon, Japan).

### Cytoskeleton staining

To examine how various hydrogel ratios affect the morphology of BMSCs, we employed fluorescein isothiocyanate (FITC)-labeled phalloidin (Solarbio, China) for cytoskeletal staining and 4′,6-diamidino-2-phenylindole (DAPI) staining solution (Solarbio, China) for nuclear visualization. On the seventh day of culturing the cells, the medium was discarded from the wells, which were then washed three times with PBS. Afterward, the cells were fixed with 4% paraformaldehyde for 30 minutes. This was followed by permeabilization for 15 minutes using 0.5% Triton X-100 (Solarbio, China). The cytoskeleton was then stained with FITC-labeled phalloidin at the recommended concentration and incubated for 12 hours at 4°C in darkness, after which the wells were rinsed with PBS. For nuclear staining, DAPI solution was applied for 10 minutes, followed by an additional wash with PBS before examination under a laser confocal microscope (Nikon, Japan).

### Senescence-associated-β-galactosidase (gal) assay

On culture days 7, 14, and 21, BMSCs cultivated in both 2D and 3D formats underwent digestion, resuspension, and were plated in six-well plates at a density of 1.0 × 10^5^ cells per well, followed by a 12-hour incubation to allow for cell adhesion. The cells underwent fixation in 4% paraformaldehyde for a duration of 30 minutes, then were rinsed with PBS (pH 7.3). Following this, BMSCs were exposed to a freshly prepared staining solution for senescence-associated (SA)-β-gal (Beyotime, China) for an overnight period. The expression of SA-β-gal was assessed by three observers, who analyzed at least 200 cells from randomly chosen non-overlapping fields. To identify and quantify the positively stained cells, three random fields were examined under a Nikon ECLIPSE Ni microscope. This experiment was conducted three times to determine the average percentage of cells that expressed SA-β-gal.

### Real-time fluorescence quantitative polymerase chain reaction

The evaluation of stemness-related gene expression was conducted utilizing the real-time fluorescence quantitative polymerase chain reaction (RT-qPCR) method. BMSCs cultured in 2D and 3D were collected for analysis. Total RNA was extracted using a specialized reagent kit provided by Tiangen Biotech in Beijing. A UV spectrophotometer was employed to assess the concentration and purity of the RNA, which was then subjected to reverse transcription to generate cDNA using the PrimeScript™ RT-PCR Kit (TaKaRa, Japan) for PCR amplification. The design and synthesis of the PCR primers (Table 1) were carried out by Shanghai Shenggong Bio-technology Co., Ltd., To assess the mRNA expression levels of key stemness-related genes such as *SOX-2* (SRY-related HMG-box transcription factor 2), *OCT4* (octamer-binding transcription factor 4), *Nanog* and *c-myc* (cellular myelocytomatosis oncogene), RT-qPCR was performed utilizing SYBR Premix Ex Taq™ on an ABI Prism7900 detection system, which is manufactured by Applied Biosystems in the USA. In order to standardize the results, *GAPDH* was used as the reference gene. Each experimental group underwent analysis in triplicate, a methodological choice made to enhance the reliability and reproducibility of the findings. The quantification of mRNA expression levels was calculated using the 2^−ΔΔ^^*Ct*^ method, allowing for a robust comparison of gene expression across different conditions.

**Table 1.** Primer sequences for RT-qPCR

Gene	Sequence (5′–3′)
*SOX-2*	F: GCTACAGCATGATGCAGGA
	R: AGCCCTGAGTGGAGAGAAGA
*OCT-4*	F: CCCTGGTATGGCTGGTAGTG
	R: AGGGTCTCCGATTTGCATATCT
*Nanog*	F: CAGCAGCTCTTCCTGATGCT
	R: CTTCCCTCTGGTCAGCTTCA
*C-myc*	F: CGTCTCCACACATCAGCACAA
	R: TGTTGGCAGCAGGATAGTCCTT
*VEGF*	F: TGTGAATGCAGACCAAAGAAG
	R: GAGGGCAGTAGCTGCGCTG
*EGF*	F: GCTGCCCTGAGAGTGTGTGA
	R: GCTGCTGGTGCTCTTCTTGT
*GAPDH*	F: CGTATCGGACGCCTGGTT
	R: AGGTCAATGAAGGGGTCGTT

### BMSC-exos extraction and characteristics

After culturing BMSCs under 2D and 3D conditions for 3 days, the cells were washed three times with PBS and subsequently replaced with a complete culture medium devoid of exosomes (OriCell, China). The cells were continued to cultivate for another 48 hours before collecting the supernatant. Differential ultracentrifugation was used to extract exosomes, applying centrifugal forces of 300×*g*, 2000×*g*, 10 000×*g* and 100 000×*g* to the cell culture supernatant. Finally, the resulting particles were resuspended in 200 μl PBS. The morphology of the extracted exosomes was then assessed using a transmission electron microscope (Hitachi, Japna), which provides detailed visualization of their structure. To confirm the identity of the exosomes, specific surface markers known to be associated with them—CD9, CD81 and TSG101—are validated using Western blot analysis. Furthermore, the size and concentration of the BMSC-exos are quantitatively assessed using the NanoSight NS500 device (Malvern Instruments, UK). The nanoparticle tracking analysis (NTA) software associated with this instrument allows for accurate determination of exosome characteristics. To standardize and eliminate any discrepancies arising from variations in cell numbers across different experimental groups, the total number of BMSC-exos is normalized by dividing by the number of BMSCs. This calculation provides the average number of exosomes secreted per BMSC, ensuring that each experimental group is analyzed consistently, with each group undergoing assessment in triplicate [27].

### Cellular uptake of BMSC-exos

Human umbilical vein endothelial cells (HUVECs) were cultured in eight-well chamber slides at a density of 1.0 × 10^4^ cells per well. These slides were then placed in a cell culture incubator for overnight incubation to promote cell attachment and growth. BMSC-exos were obtained from 2D culture (2D group) and 3D culture (3D group). To track and analyze these exosomes, a specific membrane labeling kit provided by Dojindo (Japan) was employed, adhering to the instructions provided by the manufacturer. These labeled BMSC-exosomes were then resuspended within a complete medium free of exosomes. Next, 300 μl of the medium containing labeled exosomes was added to each well and incubated in darkness for a duration of 12 hours. After performing three washes with PBS, the cells were fixed, permeabilized, and subsequently stained with DAPI to visualize the nuclei. The stained cells were subsequently analyzed using a laser confocal microscope (Nikon, Japan).

### Cell migration study

The scratch healing experiment and Transwell migration assay were used to investigate how BMSC-exos influence HUVEC migration capability. HUVECs were seeded at approximately 1 × 10^5^ cells per well in a six-well plate designated for the scratch healing assay. Once the HUVECs reached 80% to 90% confluence, two intersecting linear scratches were inflicted using a 200 µl pipette tip. Cells in the control group, 2D-exos group, and 3D-exos group were cultured in DMEM medium supplemented with 1% exosome-free FBS, either devoid of exosomes or with the addition of 2D-exos (50 µg/ml) or 3D-exos (50 µg/ml), respectively. In contrast, the ECM group was cultivated in endothelial cell medium (ECM) (Cyagen, China) containing 1% exosome-free FBS. Images of wound healing were captured at 0, 12 and 24 hours and then quantified and analyzed.

The migration experiment utilizing the Transwell assay was performed with a 24-well Transwell Chamber System Assay Kit from Corning Costar (Corning, USA). The groups included the control, 2D-exos, and 3D-exos, which were maintained in DMEM complete medium without exosomes, supplemented with 2D-exosomes (50 μg/ml), or enriched with 3D-exosomes (50 μg/ml), respectively. Additionally, the ECM group was cultured in the ECM complete medium. After a 3-day incubation period, HUVECs (1 × 10^4^) from each experimental group were transferred to the upper chamber of the transwell insert and maintained in DMEM medium supplemented with 1% serum. In the lower chamber of the 24-well plate, ECM medium enriched with 5% fetal bovine serum served as a chemoattractant. Following a 12-hour incubation, the cells were fixed using 4% paraformaldehyde for 30 minutes, rinsed with PBS and then stained for 10 minutes using a crystal violet solution. The HUVECs that had migrated to the underside of the insert were observed with a Nikon DS-Ri2 microscope, where three random fields of view per well were utilized for the counting and analysis of the migrated cells. Each group included three replicates, and the entire experiment was conducted on three separate occasions.

### Tubule formation assay

In each individual well of a 96-well plate, 50 μl of Matrigel (Beyotime, China) containing 33% DMEM medium was introduced and subsequently incubated in an incubator for 30 minutes to facilitate gelation. HUVECs underwent serum starvation in DMEM medium lacking FBS for a duration of 12 hours. After this period, the cells were plated on a Matrigel-coated surface at a density of 1 × 10^4^ cells per well. The experimental groups included the control group, the 2D-exos group, and the 3D-exos group, all of which were cultured in DMEM medium supplemented with 1% FBS. The control group did not receive any exosome supplementation, while the 2D-exos and 3D-exos groups were administered with 50 μg/ml of their respective exosomes. The ECM group was cultured in the ECM medium with 1% FBS. Following incubation for 6 hours, cells were subjected to LiveDye staining, and images were acquired with a fluorescence microscope (Nikon, Japan) for further analysis using the ImageJ angiogenesis analyzer plugin. The comparison of junctions and total segment lengths was conducted among the different groups.

### Construction of a full-thickness skin wound model

Eight-week-old male Sprague–Dawley (SD) rats (250 ± 20 g) were sourced from Sibeifu Biotechnology Co., Ltd in Beijing, China, with a sample size of 15. All animal procedures were approved by the Animal Ethics Committee of PLA General Hospital (Approval No. 2022-X18-136) and conducted in accordance with the guidelines for the Care and Use of Laboratory Animals. Anesthesia for the rats was induced with isoflurane, delivered through inhalation, and their dorsal fur was shaved off using an electric shaver. This was followed by the use of Veet cream for hair removal. In each rat, four full-thickness circular skin defects with a diameter of 10 mm were established on the dorsal surface. The group designated as the control received treatment with Vaseline gauze dressing, whereas the GelMA, 2D-exos, and 3D-exos groups received treatments involving 100 μl of GelMA, 100 μl of GelMA combined with 2D-exosomes (50 μg/ml) and 100 μl of GelMA with 3D-exosomes (50 μg/ml), respectively, to cover their wounds. Following a curing process under UV light for 30 seconds, Vaseline gauze dressing was subsequently applied. Wound assessments, including photographs, were conducted on the 3rd, 7th and 14th days post-surgery, and the area of the wounds was quantified using ImageJ software. The rats were euthanized on the 3rd, 7th and 14th postoperative days to facilitate additional experiments.

### Histology analysis

Initially, skin samples were fixed in 4% paraformaldehyde. Following this, they underwent dehydration using a series of ethanol and xylene concentrations. The samples were subsequently embedded in paraffin and cut into sections measuring 4 μm in thickness. Staining of these sections was performed utilizing hematoxylin-eosin (HE) and Masson’s trichrome to facilitate histological analysis. Finally, imaging and examination were conducted with a microscope.

### Immunofluorescence evaluation

Immunofluorescence staining was performed to investigate the development of blood vessels in wounds. Paraffin sections underwent deparaffinization, then antigen retrieval and were fixed with 3% BSA for 30 minutes. After this step, the sections were exposed to a primary antibody solution against CD31 (Proteintech, China), rinsed with PBS, and then incubated in a secondary antibody solution. Lastly, DAPI was utilized to stain the nuclei of the cells, and images of the stained specimens were obtained with a fluorescence microscope.

### Evaluation of gene expression related to vascularization

The levels of gene expression related to pro-angiogenesis in wound tissues were evaluated through the qRT-PCR method. Initially, rat wound tissues were homogenized using a lysis buffer to produce a uniform slurry. The qRT-PCR process was conducted following previously outlined protocols. The primer sequence details can be found in Table 1. Ultimately, the relative expression levels of key pro-angiogenic factors, namely *VEGF* (vascular endothelial growth factor) and *EGF* (epidermal growth factor), were determined with *GAPDH* serving as an internal control.

### RNA-sequencing analysis

In order to examine how culture dimensions affect the secretion of exosomes, we conducted transcriptome sequencing analysis on cells cultured in both 2D and 3D environments. The extraction of total RNA from the samples was performed using the TRIzol reagent (Invitrogen, USA). Afterward, the RNA was evaluated and quantified with the help of NanoDrop spectrophotometer and the Agilent 2100 Bioanalyzer (Thermo Fisher Scientific, USA). RNA libraries were subsequently generated using the Illumina^®^ NEBNext^®^ Ultra™ Directional RNA Library Prep Kit, and sequencing was conducted by a commercial service provider employing the Illumina NovaSeq™6000 platform.

The data from sequencing underwent filtering through Fastp software (version 0.14.0). Following this, the clean reads were aligned with the reference genome and mapped against the reference coding gene set using HISAT2 software (version 2.2.0). StringTie (version 2.2.1) was then employed to compute the gene expression levels. To assess differential expression, Limma (version 3.32.10) was used, applying the criteria of |log2FC| ≥ 1 and *P*≤0.05 for identifying differentially expressed genes (DEGs). To further elaborate on the biological implications of the identified DEGs, enrichment analyses were performed, focusing on Gene Ontology (GO) and the Kyoto Encyclopedia of Genes and Genomes (KEGG). These analyses were executed using the R package clusterProfiler, which enabled us to evaluate the functional categories and pathways that are significantly represented among the DEGs. Each experimental group included three biological replicates.

### Statistical analysis

The results of the study are shown as mean values ± standard deviation. The comparison between the two groups was performed using the unpaired Student’s *t*-test. When multiple comparisons were necessary, one-way analysis of variance along with Tukey’s post hoc test was applied. Throughout the analysis, statistical significance was established at a threshold of *P* < 0.05, which was consistently applied across all evaluations.

## Results

### Optimal GelMA concentration for 3D cultivation of BMSCs

**Figure 2.**
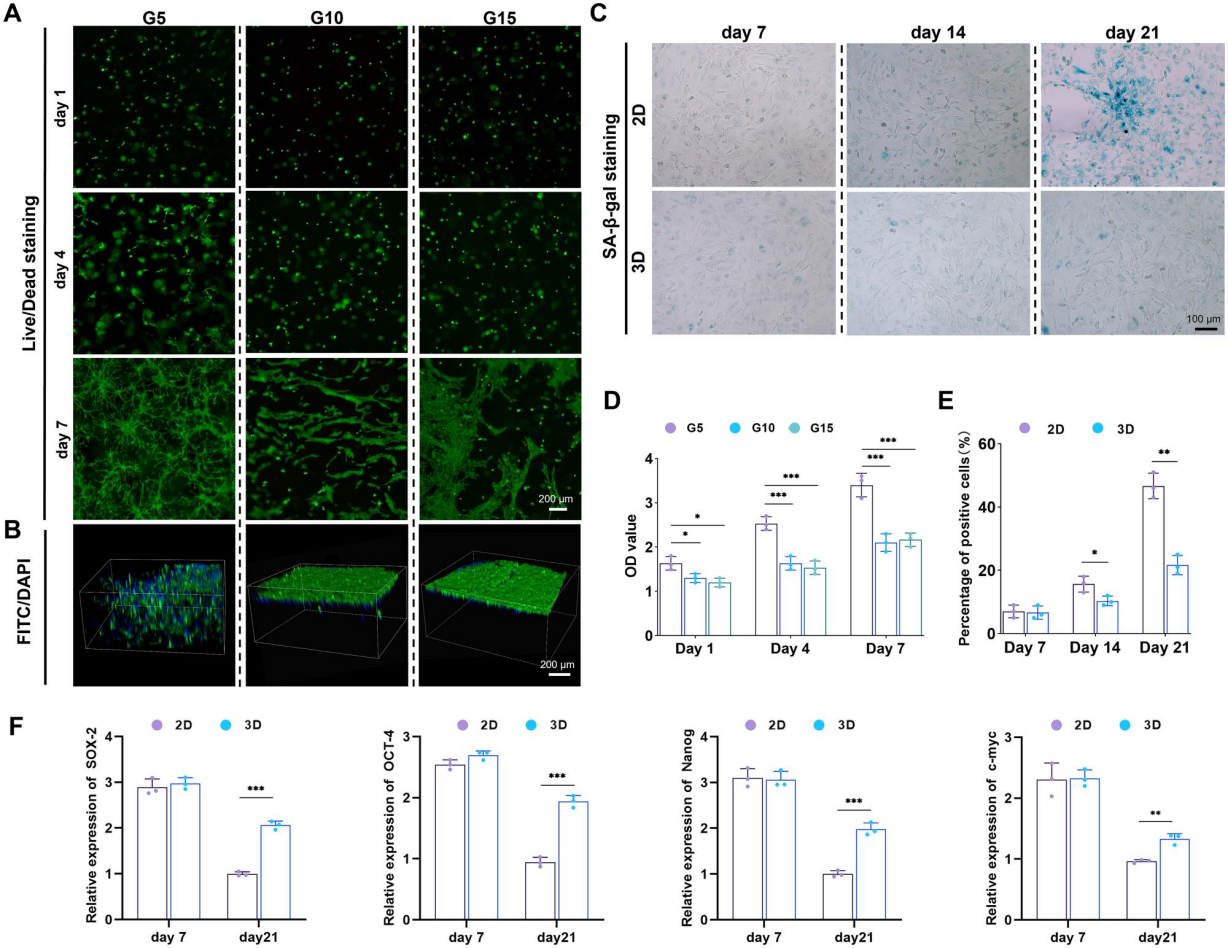
The impact of hydrogel on the proliferation, morphology and senescence of BMSCs. (**A**) Live/dead staining of BMSCs in different groups (green: live cells; red: dead cells). (**B**) Cytoskeleton staining of BMSCs in different groups (F-actin was stained with FITC-phalloidin, and the nucleus was stained with DAPI, enabling visualization of the cellular structural components). (**C**) SA-β-galactosidase staining of BMSCs in 3D culture and 2D culture. (**D**) Proliferation rate of BMSCs in different concentrations of GelMA. (**E**) Proportion of positively stained cells in SA-β-galactosidase staining in 3D culture and 2D culture. (**F**) Expression of Stemness-related genes in 3D culture and 2D culture (*P < 0.05; **P < 0.01; ***P < 0.001; ****P < 0.0001).

The live-dead staining results (Figure 2A) indicated that GelMA at different concentrations demonstrated excellent biocompatibility, with cell viability exceeding 95%. On the fourth day, the growth morphology of BMSCs in the G5 group demonstrated an expansion, whereas the BMSCs in the other two groups continued to maintain a spherical shape. By the seventh day, it was observed that cells within the G5 group grew in an intertwined manner resembling shrubs, whereas BMSCs within the hydrogels of the other two groups remained spherical. However, some BMSCs on the surface of these hydrogels displayed a flattened growth similar to 2D culture. Further staining of the cellular cytoskeleton (Figure 2B) confirmed that BMSCs in the G5 group were thriving within the hydrogel with interconnected between them. In contrast, the majority of BMSCs in the G10 group predominantly grew on the surface of the hydrogel, with only a minimal number of unextended spherical cells observed within its interior. BMSCs in the G15 group almost entirely grew on the hydrogels' surfaces, suggesting that this concentration of GelMA may not be suitable for 3D cultivation of BMSCs. The findings from the CCK8 assay (Figure 2D) reveal that the cell proliferation rate in the G5 group is considerably greater than that in the other two groups. This finding aligns with the outcomes of the live/dead staining, suggesting that a 5% concentration of GelMA is appropriate for the 3D culture of BMSCs.

### 3D culture retards the aging process of BMSCs

Due to the upregulation of SA-β-gal expression in senescent cells, staining for SA-β-gal can result in these aging cells appearing blue. As expected, a small number of blue-stained BMSCs were observed in both homogeneous groups on day 7 (Figure 2C). Over time, the quantity of blue-stained BMSCs rose in both groups. Notably, by day 14, the senescence was more evident in the cells cultured in 2D compared to those in 3D. By day 21, the majority of BMSCs in the 2D culture displayed blue staining, whereas the fraction of blue-stained BMSCs in the 3D group was considerably less than that in the 2D group, a difference that reached statistical significance (Figure 2E). A quantitative analysis was conducted to examine the variations in mRNA levels of genes related to stemness in BMSCs across the two culture dimensions (Figure 2F). Consistent with expectations, the expression levels of genes associated with stemness, such as *SOX-2*, *OCT-4*, *Nanog* and *c-myc*, demonstrated a downward trend over the culture period. At the 21-day mark, the expression of these stemness-associated genes in BMSCs cultured in 2D was significantly lower than that observed in those cultured in 3D.

### 3D culture enhances the secretion of BMSC-exosomes

**Figure 3.**
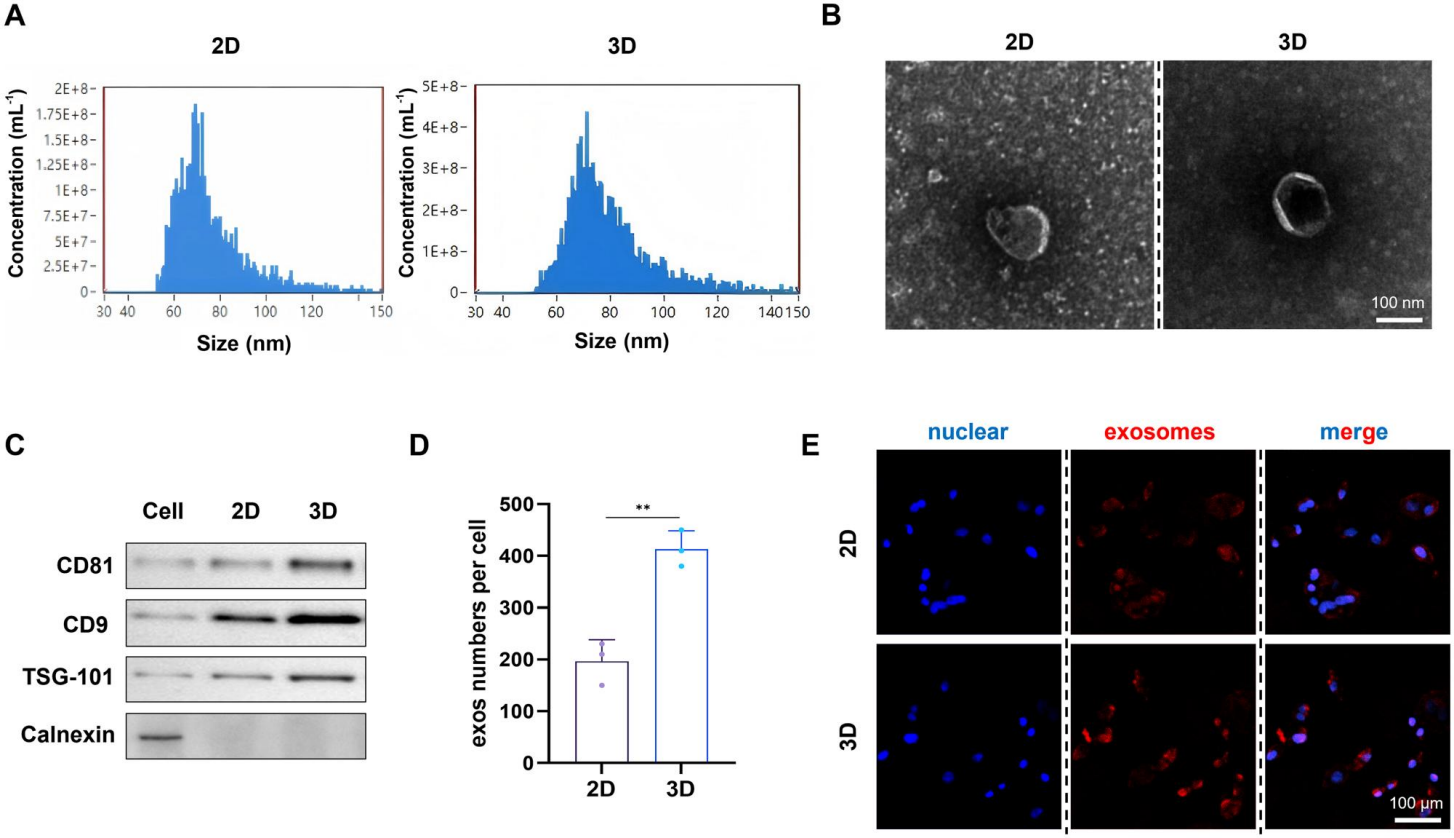
Extraction and identification of BMSC-exos. (**A**) Representative size distribution of BMSC-exos in 2D and 3D groups. (**B**) TEM images of BMSC-exos secreted in 2D and 3D groups. (**C**) The surface markers of BMSC-exos in 2D and 3D groups. (**D**) The quantity of BMSC-exos secreted by each cell in 2D and 3D groups (**P < 0.01). (**E**) Fluorescence images of cell uptake of BMSC-exos from 2D and 3D groups.

The analysis of NTA revealed that the particle size distribution in both groups of BMSC-exos conformed to the typical characteristics of exosomes (Figure 3A). TEM results revealed that both groups of BMSC-exos exhibited typical cup-shaped structures with no significant morphological differences (Figure 3B). Using the Western blot technique, the specific surface markers for exosomes were identified, demonstrating a positive expression of CD81, CD9 and TSG101 in both sets of BMSC-exos, whereas Calnexin was not detected (Figure 3C). These findings indicate successful extraction of BMSC-exos. Furthermore, it was observed that the production of BMSC-exos was enhanced by approximately 2.1 times in a 3D culture relative to a 2D culture (Figure 3D). Exosome uptake experiments indicated that both groups of exosomes could be internalized by HUVECs with no significant difference in uptake efficiency (Figure 3E).

### 3D culture enhanced the angiogenic capacity of BMSC-exos

**Figure 4.**
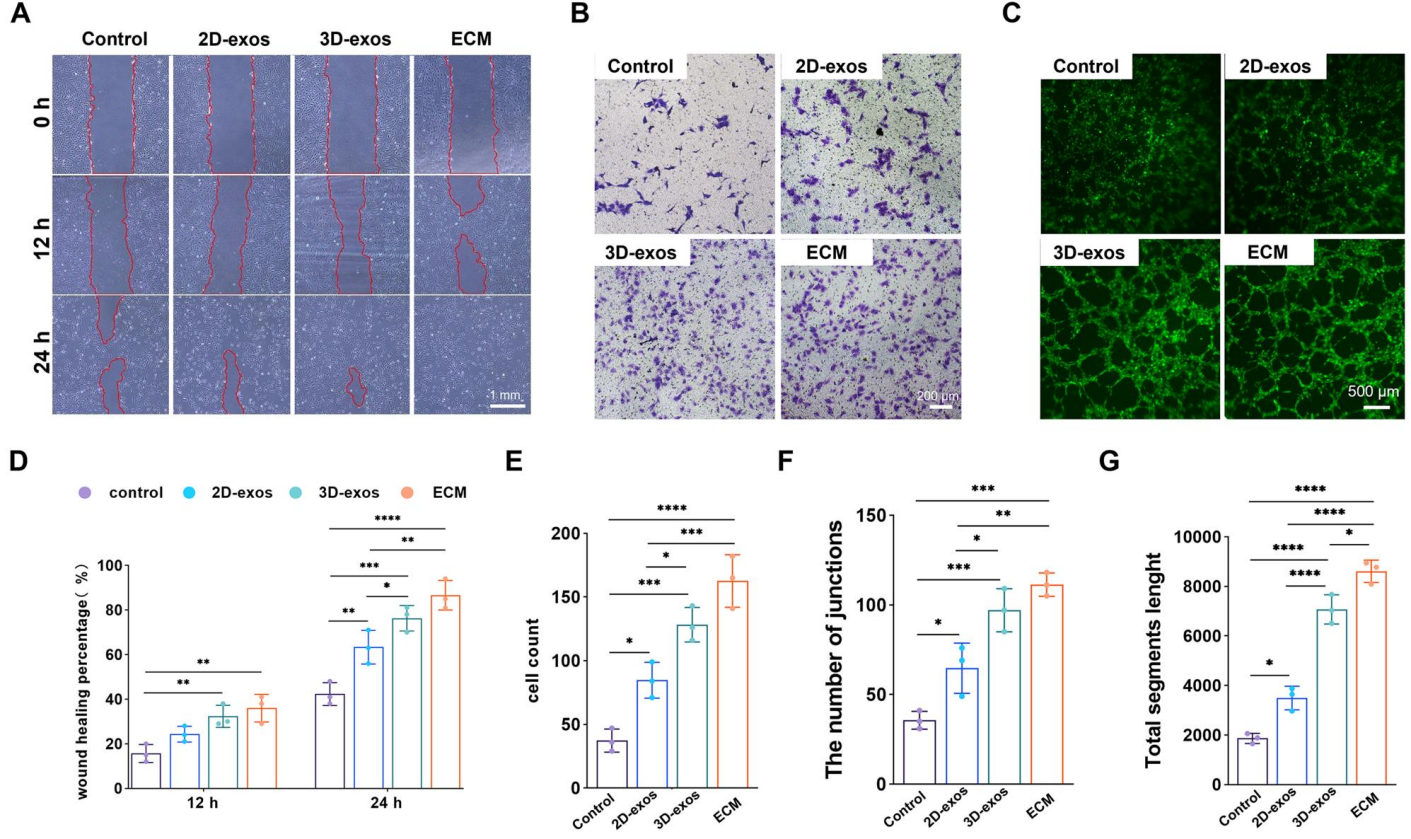
The impact of BMSC-exos on vascular endothelial cells. (**A**) Images captured from the wound healing trials at 0, 12 and 24 h. (**B**) Typical images from the transwell assay were performed at 12 h. (**C**) Typical images from the tube formation assay after 12 h. (**D**) Quantitative analysis of the percentage of wound healing observed in the experiment. (**E**) Quantitative evaluation of the cell counts in the transwell assay. (**F**, **G**) Quantitative assessment of junctions and segment length in tube formation assays (*P < 0.05, **P < 0.01, ***P < 0.001, ****P < 0.0001).

The experiment on scratch healing was performed to evaluate how BMSC-exos influence the migratory capacity of HUVECs (Figure 4A). The findings indicated that integrating BMSC-exos, in comparison to the control group, might enhance the rate of wound healing. Furthermore, at the 24-hour mark, the group receiving 3D-exos showed a considerably greater rate of wound healing than the group with 2D-exos (Figure 4D). Additionally, the ECM group culture medium is composed of a diverse array of nutritional components and serves as the positive control group. The results demonstrate that the ECM group exhibited the highest rate of wound healing at both time points. The results from the Transwell migration assay were also comparable (Figure 4B), suggesting that incorporating BMSC-exos promotes the migration of HUVECs (Figure 4E). Moreover, 3D-exos exhibited a stronger capacity to promote angiogenesis compared to 2D-exos. The Tubule formation assay was utilized to evaluate the pro-angiogenic potential of BMSC-exos (Figure 4C). The findings demonstrate that both the 3D-exos group and ECM group displayed significant tube formation, whereas the 2D-exos group exhibited a medium level of tube formation. In contrast, the control group demonstrated minimal characteristic tube formation (Figure 4F and G). The findings indicate that BMSC-exos possess the capacity to promote angiogenesis, with the 3D-exos exhibiting a more potent pro-angiogenic effect than the 2D-exos.

### 3D-exos promote full-thickness wound healing

**Figure 5.**
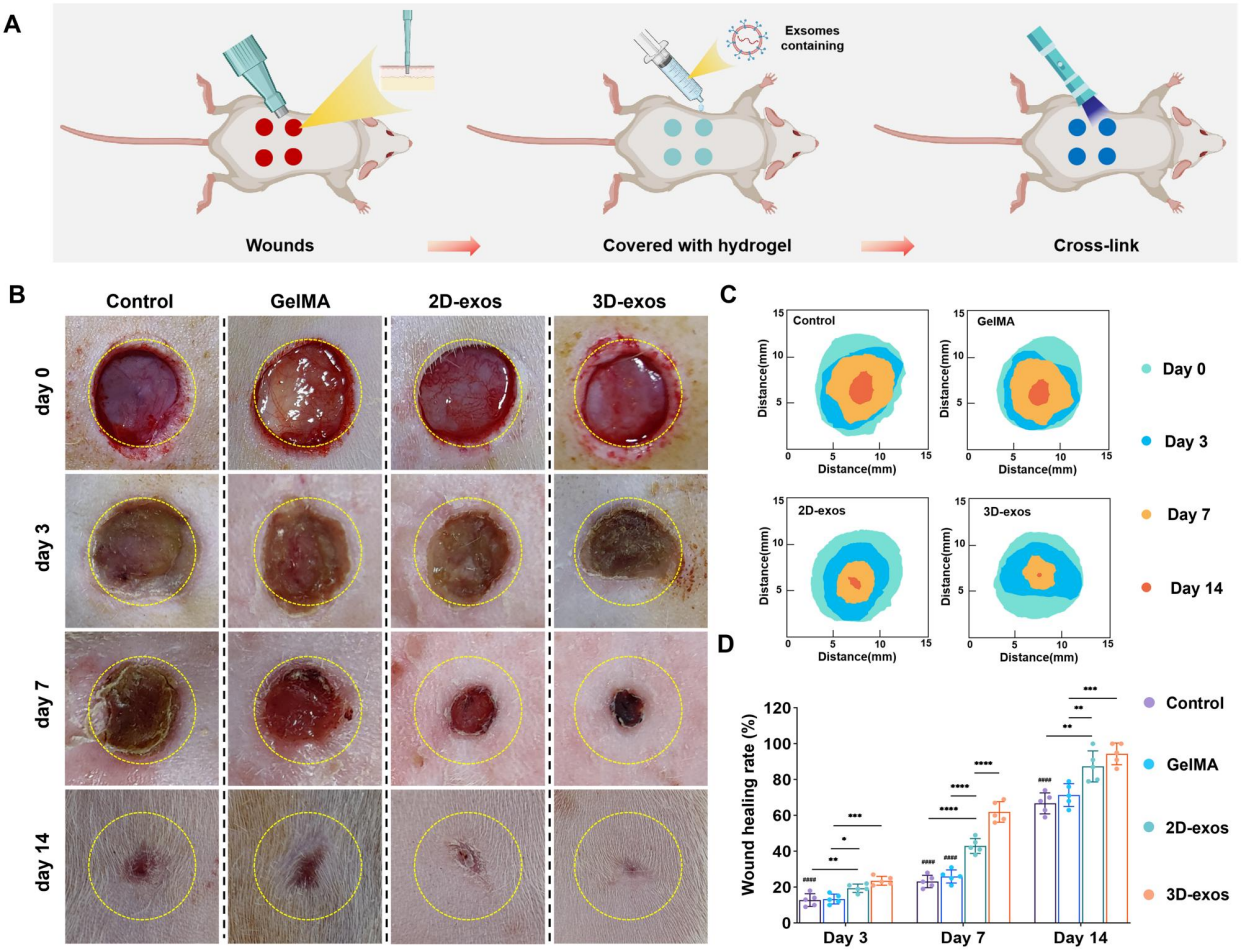
General observation of the promoting effects of BMSC-exos from different groups on wound healing. (**A**) Schematic diagram of the main process of the *in vivo* experiments. (**B**) Depictions showing the healing progression of wounds in rats following various treatments. The circle represents a diameter of 10 mm. (**C**) A diagram illustrating the status of wound healing at various time points across each group. (**D**) A quantitative assessment of wound healing across distinct time intervals (*P < 0.05, **P < 0.01, ***P < 0.001, ****P < 0.0001, ^####^comparison to 3D-exos groups *P* < 0.0001).

Additional studies were conducted to assess the therapeutic effects of BMSC-exos on the restoration of complete-thickness skin wounds using *in vivo* experiments (Figure 5A). On postoperative days 3, 7 and 14, the groups receiving 3D-exos and 2D-exos demonstrated significantly higher rates of wound healing compared to the control and GelMA groups, suggesting that BMSC-exosomes facilitate the healing process (Figure 5B and C). On the seventh day post-surgery, the healing rate of wounds in the 3D-exos group was significantly higher than that observed in the 2D-exos group. By the 14th postoperative day, the healing rates in both groups receiving exosome therapy exceeded 80%, with no statistically significant difference between them (Figure 5D). This indicates that 3D-exos may possess a superior capability of enhancing early wound healing when compared to 2D-exos.

**Figure 6.**
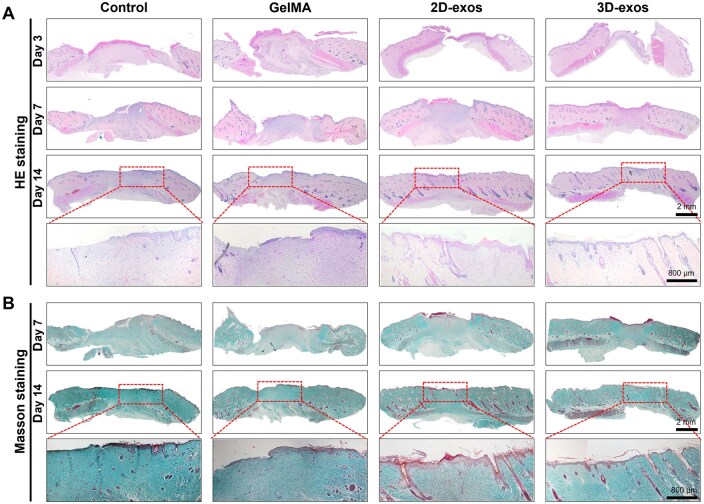
Histological evaluation of the wound-healing process. (**A**) HE staining images of skin wounds in different groups. (**B**) Masson trichrome staining images of skin wounds in different groups.

To delve deeper into the impact of BMSC-exos on wound healing, H&E staining along with Masson's trichrome technique was employed to assess the wound healing process. On the third day, varying degrees of wound healing were observed among the groups, indicating that all rats exhibited normal tissue repair capabilities. By the seventh day, it was observed that the thickness of the granulation tissue in the 3D-exos group exceeded that of the other groups. Furthermore, by the 14th day, skin appendages were seen in the 3D-exos group. In contrast, a limited number of skin appendages were noted in the 2D-exos group, while no such structures were detected in the control group and GelMA group (Figure 6A). Additionally, Masson's staining was conducted on each group to assess collagen deposition. Collagen, a vital component of the dermis, plays a pivotal role in wound healing. Increased collagen levels at the wound location facilitate faster regeneration. By days 7 and 14, the 3D-exos group showed a more extensive area of collagen accumulation at the wound site, characterized by dense collagen fibers (Figure 6B). All of these findings indicate that 3D-exos possess superior abilities in promoting wound healing.

**Figure 7.**
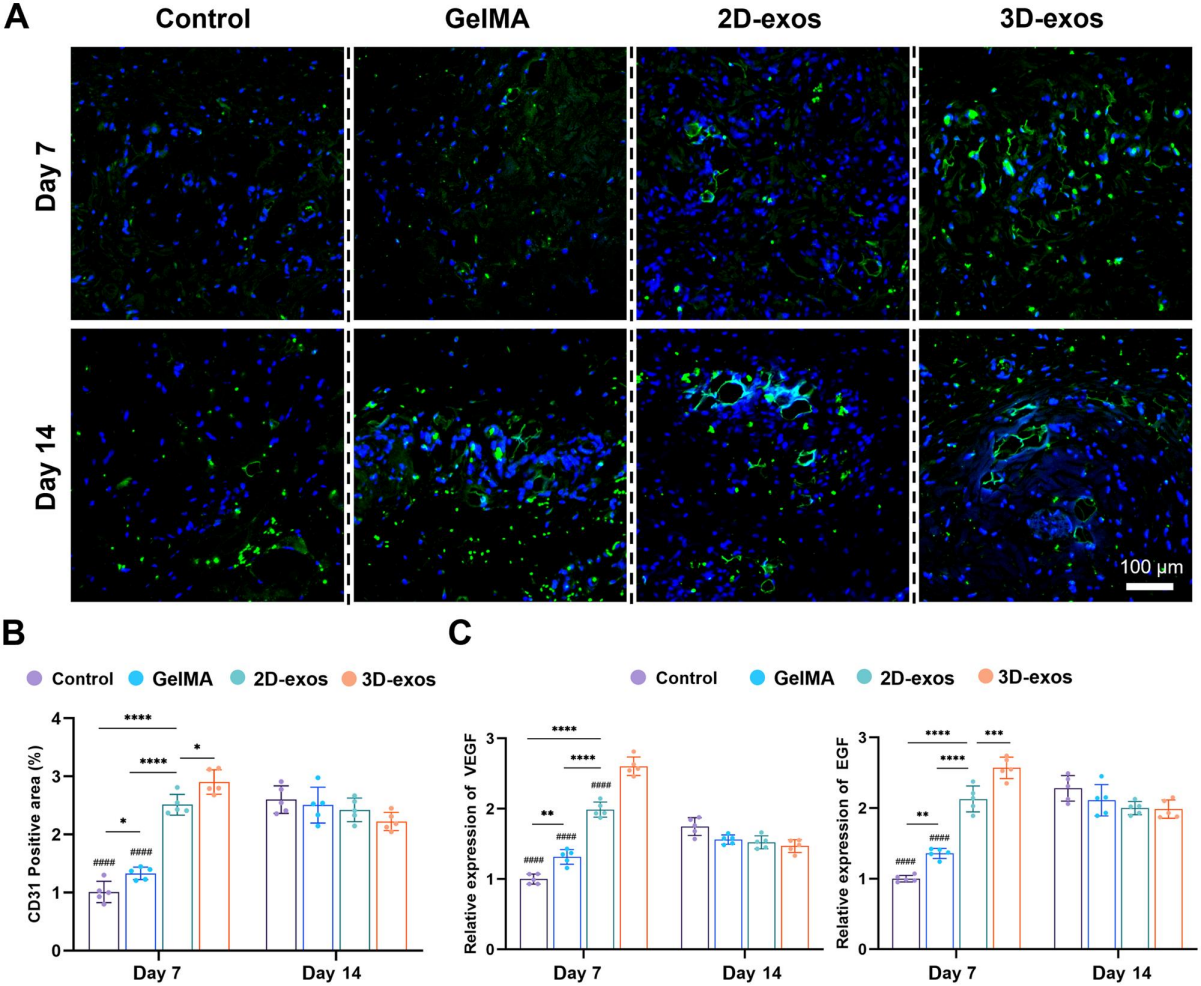
Assessment of angiogenesis in the wound. (**A**) Immunofluorescent staining of CD31 in full-thickness skin wound of rats treated with different interventions. (**B**) Quantification of the stain intensity of CD31. (**C**) Analysis of pro-angiogenic factor expression in the peri-wound region (*P < 0.05, **P < 0.01, ***P < 0.001, ****P < 0.0001, ^####^comparison to 3D-exos groups *P* < 0.0001).

Immunofluorescence staining was performed on the wound tissue, revealing a significantly larger area of CD31 positivity in the groups treated with exosomes compared to the GelMA and Control groups on the seventh postoperative day (Figure 7A). The 3D-exos group exhibited the largest fluorescent area. By postoperative day 14, a notable increase in the CD31-positive area was observed in both the GelMA and Control groups, comparable to the levels found in the 3D-exos and 2D-exos groups (Figure 7B). qRT-PCR results demonstrated higher expression of pro-angiogenesis genes in both 3D-exos and 2D-exos groups on postoperative day 7, with the 3D-exos group showing the most significant expression. However, by day 14, this difference had disappeared.

### Mechanism of 3D cultivation promoting the secretion of BMSC-exos

**Figure 8.**
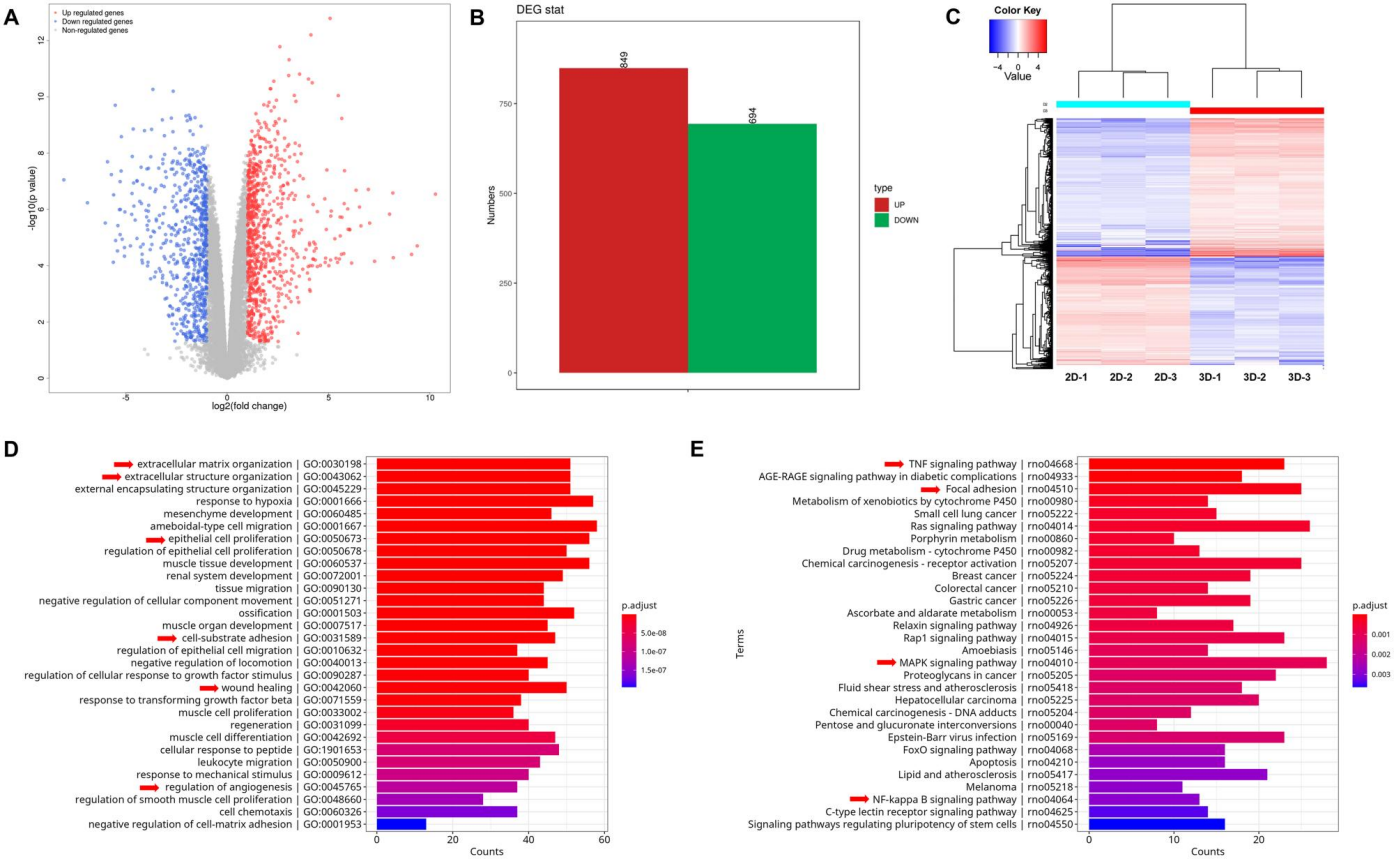
Transcriptome sequencing and bioinformatics analysis results. (**A**) Volcano plot of DEGs. (**B**) Statistical analysis of differential gene expression. (**C**) Cluster diagram of DEGs. (**D**) GO enrichment assessment for DEGs. (**E**) KEGG enrichment evaluation for DEGs.

To investigate the mechanism by which 3D culture promotes exosome secretion, we utilized RNA sequencing to examine changes in mRNA levels of BMSCs under 2D and 3D culture conditions. The analysis of mRNA sequencing revealed a significant quantity of DEGs, with 849 genes exhibiting upregulation and 694 showing downregulation (Figure 8A and B). The clustering heatmap of DEGs distinctly illustrates the separation between the 3D group and the 2D group (Figure 8C), thereby validating the reliability of the differential expression analysis results. The GO enrichment analysis indicates that these DEGs are primarily enriched in biological processes related to the extracellular matrix, such as extracellular matrix organization, extracellular structure organization, and cell-substrate adhesion (Figure 8D). Additionally, they are associated with biological processes involved in tissue repair, including regulation of epithelial cell proliferation, wound healing, and regulation of angiogenesis. KEGG enrichment analysis suggests that DEGs are primarily involved in signaling pathways including tumor necrosis factor (TNF) signaling pathway, Focal adhesion, MAPK signaling pathway, and NF-kappa B signaling pathway (Figure 8E).

## Discussion

BMSCs are essential in enhancing wound healing by directly mending impaired structures via cellular differentiation [12]. Moreover, they promote neovascularization and re-epithelialization, modulate the local immune environment, and mobilize resident stem cells, which contribute to wound healing [28, 29]. Nevertheless, the survival rate of BMSCs post-implantation is low, and their lifespan is brief [30, 31]. Consequently, they face challenges in completing self-proliferation and differentiating into specific tissue cells. Furthermore, the microenvironment of surrounding lesions does not support the survival and retention of BMSCs [16], thus further limiting their therapeutic effectiveness. Exosomes-based cell-free therapy not only demonstrates similar therapeutic effects as BMSCs but also overcomes various shortcomings associated with MSC transplantation [32]. This method has become an important area of investigation within the realm of treating wound healing [6, 17, 33]. However, the current clinical application of exosomes faces two major challenges: insufficient yield and limited reparative capacity [34]. Various chemical methods have been explored to increase exosomes secretion and enhance their functionality [34–36], but concerns about the safety of these methods still exist. In comparison, physical methods are viewed as safer. At present, enhancing exosome production through the alteration of the cell culture environment has become a significant area of investigation. Unlike the physiological conditions that allow for 3D growth, conventional 2D cell culture restricts cell growth by anchoring cells to the dish's surfaces. This limitation impacts both intercellular and cell–matrix interactions, hindering the ability of cells to accurately replicate biological functions *in vivo* [19, 25]. Consequently, this may compromise the characteristics of exosomes produced. In recent times, a significant amount of studies have shown that 3D culture can boost the release of exosomes and modify their cargo makeup, consequently improving their effectiveness [21, 37–42].

Although there is a wide variety of existing three-dimensional culture methods, their inherent limitations impede their efficacy in the production of exosomes. For instance, bioreactor-based 3D cultures require specialized equipment, which raises operational thresholds and increases funding expenditures. Furthermore, the complexity of such apparatus can elevate the risk of cellular contamination [43]. In contrast, low-attachment spheroid culture is relatively straightforward to implement; however, it does not allow for precise control over cell numbers within each spheroid, thereby complicating experimental reproducibility [44]. The hanging drop method exhibits good reproducibility; nevertheless, it involves more intricate procedures when changing media or extracting cells for subsequent analyses. Additionally, in magnetic levitation methods, the medium remains stagnant, which may lead to uneven nutrient supply and inadequate removal of metabolic waste products factors that could adversely affect long-term spheroid cultivation [19]. A 3D culture system constructed from artificially synthesized hydrogels can simulate various aspects of the physiological microenvironment of cells and can be used to study the various physiological functions of cells [23]. GelMA hydrogel is cost-effective and readily available, facilitating 3D cultivation without the necessity of specialized equipment, thereby reducing barriers to the adoption of 3D culture techniques [25]. Moreover, this method is straightforward to implement and does not significantly elevate the risk of cellular contamination. Furthermore, GelMA synthesis technology is well-established and exhibits stable properties [26], thereby imparting a high degree of reproducibility to this 3D culture method. Additionally, the porous characteristics of GelMA ensure adequate nutrient supply while allowing for the timely removal of metabolic waste products. These advantages make it promising to use GelMA-based 3D culture systems for large-scale production of exosomes. In this study, we utilized GelMA hydrogel to establish a 3D culture system. Our findings indicate that a concentration of 5% w/v GelMA-30 was the most suitable for promoting the growth of BMSCs. This concentration not only facilitates robust proliferation but also results in a denser cell morphology resembling thicket-like structures. Conversely, higher concentrations of GelMA inhibited cell proliferation within the hydrogel and led to a morphologically spherical appearance with a low mass/nuclear ratio, consistent with previous reports [26]. These changes were correlated with an enhancement in the mechanical properties of hydrogels. Beyond their impact on cell proliferation and morphology, 3D culture also slows down cell aging [45, 46]. Yin *et al*. [22] discovered that the water-based hydrogel 3D culture system can alleviate senescence-related alterations in ADMSCs. Meanwhile, Miceli *et al*. [47] observed an enhancement in the expression levels of critical stem cell markers in MSCs cultured within a 3D setting. Similarly, our research results demonstrate that the GelMA-based 3D culture system can delay BMSC aging. 2D cell culture requires frequent passaging, which exposes cells to the effects of trypsinization and centrifugation [48]. Additionally, substrate attachment in 2D culture often leads to cellular senescence and reduced differentiation potential [47]. In contrast, 3D cell culture avoids these issues and thus delays cellular senescence. We further investigated the secretion of exosomes, and in line with previous findings, we observed that 3D culture promoted an increased secretion of exosomes from individual cells. Therefore, the GelMA-based 3D culture method not only simplifies the time-consuming and labor-intensive cell passage process but also allows cells to maintain their vitality and increases the yield of single-cell exosomes. This suggests that GelMA-based 3D culture is a valuable approach for the large-scale generation of exosomes.

The process of wound healing significantly relies on vascularization, which includes a complete series of repair and remodeling activities [14]. The development of new blood vessels is essential for optimal functioning since it delivers adequate oxygen and nutrients to the injured region, which in turn promotes the activities of cells involved in repair and encourages tissue regeneration [15]. Exosomes have been demonstrated to enhance wound healing by accelerating wound vascularization [17, 49]. Research carried out by Hu *et al*. [50] has shown that exosomes can improve the vascular regeneration capabilities of HUVECs by stimulating the PI3K/AKT/eNOS signaling pathway. Consequently, this promotes faster healing of wounds in diabetic conditions. Zhu *et al*. [20] found that exosomes produced from 3D culture considerably boosted the proliferation and migration of HUVECs, as well as their angiogenic potential. Our research also found that BMSC-exos possess the capability to enhance the angiogenic characteristics of HUVECs, a capability that can be further amplified by employing a 3D culture system utilizing GelMA. We have also validated these findings through animal experiments. Because of the rapid clearance ability of the circulatory system, exosomes face challenges in maintaining prolonged activity at specific locations to repair targeted tissues [51]. To prolong the therapeutic impact of BMSC-exos on wound healing, we encapsulated them into a 10% GelMA hydrogel. GelMA is in a fluid state prior to curing, exhibiting high flexibility that allows it to easily and completely cover wounds [52]. After photocrosslinking, GelMA photocures and forms bioadhesives that adhere seamlessly to the wound surface. These bioadhesives play a crucial role in hemostasis by effectively sealing off the wounded area and preventing the entry of external bacteria. Ensuring proper healing and minimizing the risk of infection are especially crucial when treating acute wound defects. Our research findings demonstrate that GelMA effectively covers the wound surface. On the seventh postoperative day, we observed that exosomes significantly promoted wound healing, with the highest healing rate noted in the 3D-exos group. CD31 staining and PCR results indicated that this enhanced repair capability was associated with improved vascularization at the wound site. By the 14th postoperative day, all groups had achieved a relatively high wound healing rate; nonetheless, the findings from HE and Masson staining showed that the quantity of newly formed hair follicles and glands in the 3D-exos group was substantially greater than what was seen in the other groups. This finding indicates that tissue repair quality in the 3D-exos group surpassed that of the other groups. Additionally, throughout this timeframe, the expression levels of genes related to angiogenesis in the 3D-exos group showed a significant decrease in comparison to earlier measurements. This observation aligns with findings from earlier research [20, 53]. We propose that early in wound healing, exosomes promote angiogenesis, facilitating a quicker transition of wounds into the proliferative phase. By day 14, as wound healing progressed into the remodeling stage, a significant reduction in vascular markers was observed in the 3D-exos group. Conversely, vascular markers in the control group increased relative to prior measurements, indicating that this group remained within the proliferative phase and demonstrated delayed tissue repair. This result indicates that GelMA loaded with 3D-exosomes holds considerable promise in the field of wound healing.

Understanding the mechanisms through which 3D culture affects exosome secretion and function is essential for its clinical applications. The research conducted by Yuan *et al*. [21], demonstrated that 3D aggregate culture significantly influences the expression of cellular mRNA. They suggested that enhanced intercellular interactions and the increased presence of extracellular matrix may be critical factors facilitating the secretion of extracellular vesicles in 3D aggregate cultures. Our research findings also indicate that 3D culture based on GelMA significantly alters the gene expression profile of BMSCs. The DEGs are primarily enriched in biological functions related to extracellular matrix organization and extracellular structure organization, suggesting that the enhanced secretion of exosomes in this 3D culture may be associated with changes in the extracellular matrix. Furthermore, we observed that the DEGs are closely associated with biological functions such as the regulation of epithelial cell proliferation, wound healing, and regulation of angiogenesis. We hypothesize that 3D culture can activate the repair capabilities of cells and may exert these functions by altering the contents of exosomes. This may explain why exosomes derived from 3D cultures exhibit enhanced tissue repair capabilities [38, 49]. Nevertheless, further research is required to unravel the underlying mechanisms involved. The secretion process of exosomes is regulated by various signaling pathways [54]. Nakao *et al*. [55] discovered that stimulation with TNF-α significantly increases the release of exosomes from MSCs. Wu *et al*. [56] found that pretreatment with TNF-α triggers the activation of the PI3K/AKT signaling pathway in MSCs, which in turn enhances exosome release. Our research results indicate that DEGs are enriched in the TNF signaling pathway, suggesting that GelMA-based 3D culture may facilitate exosome release through activation of this pathway. Additionally, a study has indicated a reduction in F-actin expression in MSCs cultured within 3D environments. This reduction leads to a relaxation of cytoskeletal tension, consequently fostering a more advantageous setting for exosome synthesis and release [57]. Through KEGG enrichment analysis, we discovered that DEGs were significantly enriched in focal adhesion pathways, implying that the mechanism by which GelMA-based 3D culture promotes exosome release may be associated with cytoskeletal remodeling. Furthermore, earlier research has demonstrated that the MAPK/ERK signaling pathway plays a role in regulating the release of exosomes [58]. Our study also demonstrated the enrichment of DEGs within this pathway. Although determining the specific roles of individual signaling pathways throughout biological processes based solely on transcriptomic analyses presents challenges, our findings provide valuable insights into further exploration into how 3D culture enhances exosome secretion.

## Conclusion

In conclusion, we have established a straightforward and effective 3D culture system for the growth of BMSCs utilizing GelMA. Our findings demonstrate that this 3D culture environment can alleviate the aging process of BMSCs and boost the secretion of exosomes derived from these cells. Moreover, exosomes harvested from BMSCs cultured within this 3D system exhibit improved biological functions, particularly in promoting angiogenesis to facilitate wound healing. Consequently, this 3D culture method offers significant advantages by reducing both the time and economic costs associated with the production of BMSC-derived exosomes, potentially paving the way for new directions in industrial-scale exosome production in the future.
